# SurvBoard: standardized benchmarking for multi-omics cancer survival models

**DOI:** 10.1093/bib/bbaf521

**Published:** 2025-10-01

**Authors:** David Wissel, Nikita Janakarajan, Aayush Grover, Enrico Toniato, Maria Rodríguez Martínez, Valentina Boeva

**Affiliations:** Department of Computer Science, ETH Zurich, Zurich, Switzerland; Department of Molecular Life Sciences, University of Zurich, Zurich, Switzerland; Swiss Institute of Bioinformatics, Lausanne, Switzerland; Department of Computer Science, ETH Zurich, Zurich, Switzerland; IBM Research Europe, Zurich, Switzerland; Department of Computer Science, ETH Zurich, Zurich, Switzerland; Swiss Institute of Bioinformatics, Lausanne, Switzerland; IBM Research Europe, Zurich, Switzerland; IBM Research Europe, Zurich, Switzerland; Yale School of Medicine, New Haven, CT 06510, USA; Department of Computer Science, ETH Zurich, Zurich, Switzerland; Swiss Institute of Bioinformatics, Lausanne, Switzerland; Université de Paris UMR-S1016 Institut Cochin, Inserm U1016 Paris, France

**Keywords:** multi-omics, survival analysis, cancer, deep learning

## Abstract

Multi-omics data, which include genomic, transcriptomic, epigenetic, and proteomic data, are gaining increasing importance for determining the clinical outcomes of cancer patients. Several recent studies have evaluated various multimodal integration strategies for cancer survival prediction, highlighting the need for standardizing model performance results. Addressing this issue, we introduce SurvBoard, a benchmark framework that standardizes key experimental design choices. SurvBoard enables comparisons between single-cancer and pan-cancer data models and assesses the benefits of using patient data with missing modalities. We also address common pitfalls in preprocessing and validating multi-omics cancer survival models. We apply SurvBoard to several exemplary use cases, further confirming that statistical models tend to outperform deep learning methods, especially for metrics measuring survival function calibration. Moreover, most models exhibit better performance when trained in a pan-cancer context and can benefit from leveraging samples for which data of some omics modalities are missing. We provide a web service for model evaluation and to make our benchmark results easily accessible and viewable: https://www.survboard.science/. All code is available on GitHub: https://github.com/BoevaLab/survboard/. All benchmark outputs are available on Zenodo: 10.5281/zenodo.11066226. A video tutorial on how to use the Survboard leaderboard is available on YouTube at https://youtu.be/HJrdpJP8Vvk.

## Introduction

Survival analysis models for cancer research aim to predict survival-related information using data with potentially censored and truncated observations [1]. These models play a crucial role in patient risk stratification and improving treatment selection, and are gaining increasing interest from the machine learning and bioinformatics communities, and are partially being evaluated for therapeutic applications in some types of cancer [2–7].

With the advent of large-scale cancer programs such as The Cancer Genome Atlas TCGA, International Cancer Genome Consortium ICGC, and Therapeutically Applicable Research to Generate Effective Treatments TARGET, researchers have begun to incorporate multimodal omics data into their survival models [8–16], especially given the increasing importance of deep learning in bioinformatics in general and survival analysis in particular [17, 18].

In addition, multimodal cancer datasets are increasingly being used for additional tasks such as cancer tissue of origin prediction [19–21]. However, to date, most existing works have exclusively exploited the TCGA datasets due to their large size and extensive omics information, potentially increasing the risk of overfitting models to this cancer program [22–26]. Comparison of survival prediction methods across large-scale cancer programs has also been increasingly difficult due to the many divergent choices regarding data imputation, cancer types under consideration, test splits, and omics types utilized. Furthermore, while a considerable number of benchmarks have explored statistical and regression models for multi-omics integration in the cancer survival context [23, 27, 28], no comprehensive benchmark has compared neural and statistical models specifically in the multi-omics setting, except our recent work, which focused primarily on the noise resistance properties of different models, as opposed to overall performance [25].

In a pioneering study, Zhao *et al.* [27] benchmarked several feature selection and dimensionality reduction methods combined with the Cox proportional hazards model on four cancer types from the TCGA program. Although there was high variability across cancer types, the study concluded that modalities beyond clinical and gene expression did not significantly enhance prediction performance. However, this study was conducted early in the life cycle of TCGA, and it only considered a limited number of datasets and techniques. More recently, Herrmann *et al.* [23] evaluated the performance of 12 statistical multi-omics models in predicting cancer survival across 18 TCGA cancer types. The study found that while incorporating the multimodal group structure of multi-omics data resulted in better predictions, even the best-performing multi-omics models did not significantly outperform a baseline model trained solely on clinical data. It should be noted that this study excluded neural network (NN) models, now frequently employed in survival analysis [24, 26]. Furthermore, this study did not consider missing modalities or pan-cancer scenarios in the training data, which are increasingly common in NNs designed for cancer survival prediction [15, 24, 26]. [29] provided a comprehensive review and benchmarked multiple methods designed to handle partially missing modalities for a classification task on TCGA. Nießl *et al.* [30] used the benchmark design of Herrmann *et al.* [23] to illustrate the multiplicity of design options available to benchmark multi-omics survival analysis. They showed that the benchmark results could vary widely depending on the metrics, datasets, and models used.

In addition to different training scenarios and the absence of deep learning models in previous benchmarks [23], there exist few guidelines for benchmarking survival models more generally. The lack of a standardized benchmarking and experimental framework can cause overly optimistic results due to inadvertent data leakage and the numerous preprocessing options available to researchers when comparing different survival prediction methods [30, 31].

To address the current gaps in the performance evaluation of multi-omics cancer survival models and to standardize their empirical comparison, we introduce SurvBoard, a comprehensive benchmarking framework. Overall, we summarize our contributions as follows:

1. Using SurvBoard, we evaluate the predictive performance of deep learning and state-of-the-art statistical models on datasets from four cancer programs: TCGA, ICGC, TARGET, and Molecular Taxonomy of Breast Cancer International Consortium METABRIC.
2. SurvBoard allows users to train models in three different settings: standard survival analysis, survival analysis with samples for which some data modalities are missing, and pan-cancer analysis, where a model is jointly trained on multiple cancer types. We benchmark the effect of adding these training modalities explicitly.
3. We showcase the potential use of the SurvBoard platform and discuss common pitfalls in creating datasets for omics survival analysis studies using relevant examples from our four considered cancer programs.

We emphasize that while multi-omics cancer survival analysis currently has limited clinical applications, benchmarking remains essential for advancing the field. Rather than focusing solely on ranking models, our benchmark aims to identify key components that contribute to model performance. Consequently, throughout our work, we use the term benchmark to refer primarily to the overall benchmarking framework we have constructed, rather than just to the specific experiments conducted within it.

## Materials and methods

### Datasets

The SurvBoard benchmark includes a total of 28 cancer datasets from four projects: TCGA, which is arguably the largest and most commonly used database for multi-omics cancer survival analysis ($n=21$ datasets), ICGC, which encompasses and complements TCGA with additional samples from non-American studies ($n=4$ datasets), the pediatric cancer database TARGET ($n=2$ datasets), and the large breast cancer dataset METABRIC ($n=1$ dataset) (Supplementary Tables S1 and S2). All datasets from cancer programs were preprocessed according to the selection criteria highlighted in the Preprocessing section and Supplementary Methods.

Going forward, we will restrict the study to right-censoring with no truncation, which is typical of most large-scale observational cancer studies and datasets.

### Survival analysis models evaluated in the leaderboard

We evaluated twelve different approaches on SurvBoard to jumpstart the leaderboard, including two statistical methods and ten deep learning models. Our selection of statistical methods was based on the research conducted by Herrmann *et al.* [23] and Wissel *et al.* [25], who identified BlockForest [22] and PriorityLasso [32] as the leading methods for accurately predicting clinical outcomes on TCGA datasets.

From previous research on multimodal deep learning architectures for multi-omics survival analysis, the most effective models were found to be architectures based on late fusion using an arithmetic mean and intermediate fusion using concatenation [25]. Furthermore, we used two loss functions for the deep learning methods: the commonly used Cox PH partial likelihood and the Extended Hazards (EHs) likelihood, which was recently introduced in a deep learning setting [4, 33]. We only considered methods that take into account the group structure of the multi-omics data as they have been proven to be more effective than those that do not [23, 25]. In addition to these generic models, we also considered six state-of-the-art deep learning methods for multi-omics cancer survival prediction.

Thus, to seed the SurvBoard leaderboard, we conducted experiments for the twelve methods described below:

1. **PriorityLasso L1+L2** (with Elastic-net regularization), a method that orders the input modalities and sequentially uses Elastic-net-based models per modality that are carried forward via offsets into the model fit for the next modality [32];
2. **BlockForest**, a method based on random survival forests, that takes the group structure of multi-omics data into account by sampling covariates per modality (as opposed to uniformly) and considers block-specific weights when calculating the split criterion [22];
3. NN using late fusion with an arithmetic mean and with the Cox PH likelihood, **NN Cox LM** [34, 35];
4. NN using late fusion with arithmetic mean and the EHs likelihood, **NN EH LM** [4];
5. NN using intermediate fusion with concatenation and the Cox PH likelihood, **NN Cox IC** [34, 35];
6. NN using intermediate fusion with concatenation and the EHs likelihood, **NN EH IC** [4].
7. **Salmon** [11]: A deep learning method that performs intermediate fusion using concatenation. This model is trained using the Cox PH loss and leverages eigengene matrices for preprocessing before training.
8. **GDP** (Group Lasso regularized deep learning for cancer prognosis) [12]: A deep learning method that performs early fusion combined with sparse group-lasso regularization, either between gene groups or modality groups.
9. **SurvivalNet** [36]: A deep learning method that performs early fusion with the Cox PH loss.
10. **Multimodal NSCLC** [14]: A method that performs feature selection per modality based on univariate Cox PH $P$-values, followed by a denoising autoencoder. An Elastic Net Cox PH model is then trained on the resulting latent space.
11. **MultimodalSurvivalPrediction** [15]: A method that employs intermediate fusion with attention mechanisms to integrate multiple modalities, optimized using a Cox PH loss function.
12. **CustOmics** [16]: A method that uses a two-step training procedure based on hierarchical variational autoencoders for modality integration, combined with optimization using a Cox PH loss.

Further details regarding the considered models, including hyperparameter choices, and likelihood functions for likelihood-based methods can be found in Supplementary Methods.

### Considered modalities

The performance of each model was evaluated in three different scenarios: (i) on each modality individually, (ii) with clinical and gene expression data combined, and (iii) with all modalities available for that particular dataset. Notably, available modalities significantly varied across cancer programs and datasets (Supplementary Table S1).

Furthermore, for experiments where only one modality was used and no multimodal integration was required, equivalent models that did not take group structure into account were employed. For example, Elastic Net was used instead of PriorityLasso, and Survival Random Forest instead of BlockForest in the unimodal experiments. For all deep learning models, the unimodal experiments used a standard Multi-Layer Perceptron.

### Three settings for the evaluation of survival models

SurvBoard allows users to train models in three settings: standard, missing data modality, and pan-cancer.

#### Standard setting

Our first setting implements standard multi-omics survival analysis. Each model is trained and evaluated only on samples of the same cancer type, excluding all samples that do not have data for the chosen modalities for that cancer type (see Supplementary Methods).

#### Missing data modality setting

The missing data modality setting refers to the scenario in which several samples in a dataset lack data for one or more modalities, but still have data for some other modalities, in addition to survival information. This is common, e.g. in TCGA, where many patients lack protein expression reverse-phase protein arrays (RPPA) data. Thus, models that can handle samples with missing modalities benefit from an increased training set size.

A model’s ability to handle missing data allows it to train on more examples. The benefits of this additional training data can be assessed by evaluating performance on the same test set as the standard setting.

#### Pan-cancer setting

In the pan-cancer setting, models can be trained jointly on datasets from multiple cancer types. However, since not all datasets contain all modalities, models that cannot handle missing modalities cannot be trained in the pan-cancer setting when all modalities are used. Therefore, in our pan-cancer experiments, we only used clinical data and gene expression.

It is worth noting that the pan-cancer scenario only applies to the TCGA project, as other projects did not provide data that was normalized in a unified way for a pan-cancer analysis.

For the test sets, only those samples with complete data for all chosen modalities in all settings were included. The idea behind having a consistent test set is to enable a fair comparison across all model types independent of their features. By doing so, models that can handle missing modalities or those that have been trained on pan-cancer data can be compared against models trained in the standard way, allowing the inclusion of such models as baselines.

### Preprocessing

While several packages, e.g. Cerami *et al.* [37], and data sources, e.g. Weinstein *et al.* [38], allow the acquisition and usage of TCGA, ICGC, TARGET, and METABRIC datasets, preprocessing choices are left to the user, which leads to inconsistency across experiments. To enable a fair comparison of existing and new methods, SurvBoard standardizes most preprocessing choices.

#### Endpoint choice

While all of our considered cancer datasets provide multiple endpoints, e.g. Overall Survival OS, Disease Free Survival, and others, it is relatively common in the survival literature to utilize OS, as it is ubiquitously available compared to other endpoints. For example, for TCGA, the Clinical Data Resource analyzed the suitability of different endpoints for survival analysis and found that OS is the most used and generally appropriate for most datasets [39]. The only exception was the situation where progression was the event of interest, in which case the progression-free interval was recommended. We follow this broader convention and use OS as the endpoint of all datasets within our benchmark.

#### Patient cohort

We restricted our datasets to primary tumor tissue samples. We also excluded patients for whom either the event indicator or the event time was missing, as well as patients (in TCGA), for whom age at diagnosis information was missing.

#### Dataset selection

We followed a similar methodology as Herrmann *et al.* [23] and selected only datasets with at least $100$ samples and a minimum event ratio of $5 \%$ or $10$ total events, whichever was larger. This ensured that we could compute meaningful performance metrics. We only counted samples with complete modalities for dataset selection since only these were included in the test splits. Samples with missing modalities were only used as additional training data.

#### Modality selection

We chose the maximum number of modalities available for each dataset in each cancer program and excluded datasets lacking clinical data and gene expression modalities. In addition, we selected only datasets that fulfilled the criteria above for at least two omics modalities, leading to a total minimum of three modalities (at least two of which were omics) for each dataset.

#### Clinical variables

To ensure a fair comparison between different cancer programs and cancer types, we only considered standard clinical information that was available at the time of diagnosis to prevent data leakage. This included demographic data such as age and gender, and staging variables such as clinical stage. For each cancer program and dataset, we used slightly different variables in the SurvBoard framework (as outlined in Supplementary Methods and Supplementary Table S1). For TCGA, we chose not to include information specific to certain cancer types in our analysis, such as smoking history for lung cancer, to best enable the pan-cancer setting.

#### Missing values within modalities

To handle missing data, we followed a three-step procedure. First, we created a token for non-available (NA) information for categorical variables. We assumed that the missingness of categorical variables might correlate with either the target variable or other covariates, which is known as Missing Not At Random [40]. Our goal was to avoid mixing unrelated categories, so we did not use mode imputation. Second, non-categorical variables missing in more than $10 \%$ of samples in a specific dataset were excluded from that dataset. Third, non-categorical variables with missing rates less than $10 \%$ in a dataset were imputed using the median of the available samples on the full dataset, except for clinical variables, where such may not be considered reliable. Although imputation in the full dataset could lead to some information leakage, previous research has shown that it does not cause significant bias [41]. This approach was designed to remove non-model-specific preprocessing decisions from researchers’ discretion.

#### Missing modalities

To create splits for training and testing for each dataset of each cancer program, we created two sets of samples: a complete set used in the standard setting and an incomplete set that included samples with one or more missing modalities. Importantly, the incomplete set was intended only as additional training data in our benchmark, as noted in the section describing the three training settings. Within the incomplete set, NA values indicated that a particular modality was missing in a specific sample. As explained above, NA values for particular variables within available modalities were no longer present in the incomplete set as they had been imputed or removed.

#### Pan-cancer training

For the TCGA program, which provided data normalized in a pan-cancer way, we combined variables across all cancer types in the pan-cancer dataset. The variables that were not available for all cancer types were excluded. However, if a particular cancer type lacked a modality, we did not remove this modality from the pan-cancer dataset. Instead, we marked it as missing for the samples corresponding to that particular cancer type.

### Performance metrics

We measured three performance metrics in our benchmark, all of which are evaluated on the survival function level. First, we used Antolini’s Concordance (Antolini’s C) to assess the ability of each survival model to discriminate low-risk patients from high-risk patients over time [42]. Second, we evaluated the Integrated Brier Score IBS, which is a widely used measure in survival benchmarks that assesses both discrimination and calibration accuracy [43]. Third, we included the recently proposed D-Calibration D-CAL, which measures the distributional calibration of each multi-omics survival model [44]. For D-CAL, we evaluated the test statistic where lower values corresponded to a better fit (Supplementary Methods).

Antolini’s C, which is widely used in survival analysis, only considers the stratification power of a model in discriminating between high and low-risk patients. Although it is easy to interpret and handles censored data well, the accuracy in predicting the survival probability is also of considerable importance, given its implications in the clinical setting. For this reason, we included the IBS, which takes into account both discriminative and calibration abilities of a model. The IBS requires an estimation of the censoring distribution, which is commonly performed unconditionally using a Kaplan–Meier (KM) on the test set [45]. However, recent work has shown that the IBS can yield better scores for models that do not match commonly accepted notions of calibration [44]. To address this, we introduced a third metric, D-Calibration. This metric only considers the calibration power of the model and can identify systematic under- or over-estimation. However, D-Calibration relies on the binning of predictions, which may cause issues, and is still less widely used compared to the other metrics. By incorporating multiple metrics, we gain a fair overview of a model’s strengths and weaknesses, distinguishing whether it excels in discrimination, calibration, or both. This, in turn, enables users to select models best suited to their specific tasks. We highlight these aspects in our case studies.

### Validation

We implemented five-fold cross-validation, repeated five times, resulting in a total of $25$ test splits for each cancer type. To create the splits, we stratified the data by the event indicator, OS, which ensured that the event ratio in train and test folds was comparable. The incomplete modality samples were not part of the test set and were instead used only as additional training data. In the pan-cancer setting, training data from all cancer types were included in each training split.

In cases where a model encountered numerical issues or sparse methods reported a fully sparse fit as the best model, we used a KM estimator as a replacement [46]. For instance, the Lasso model has been observed to sometimes fail in very high-dimensional datasets with high multicollinearity due to numerical issues [23, 47]. We note that other choices have been explored here and that this choice may have an impact on results [23, 30]. Despite this, to enable a fair comparison and prevent gameability for future submissions to SurvBoard (e.g. by deliberately setting difficult splits to failures), we settled on the choice of a simple KM replacement (see Supplementary Methods).

### External validation

In addition to standard cross-validation within the same dataset, we also performed external validation by transferring models across datasets, for the same cancer type. Specifically, we applied model transfer for liver and pancreatic cancer data.

For each of these, we trained models on the larger dataset (TCGA liver hepatocellular carcinoma, TCGA-LIHC, and ICGC pancreatic cancer – Canada, ICGC-PACA-CA) and transferred these models to make predictions on the corresponding smaller dataset (ICGC liver cancer – Riken, Japan, ICGC-LIRI-JP, and TCGA pancreatic adenocarcinoma, TCGA-PAAD). To ensure the same features, we restricted this study to using only gene expression and clinical features that were common to the two datasets for each cancer type. In addition, expression data were quantile-normalized before splitting [48]. For each training split of the training dataset (see above), we trained a model and transferred it to the full testing dataset, where it was evaluated as described above for cross-validation.

## Results

### Benchmark design

**Figure 1:**
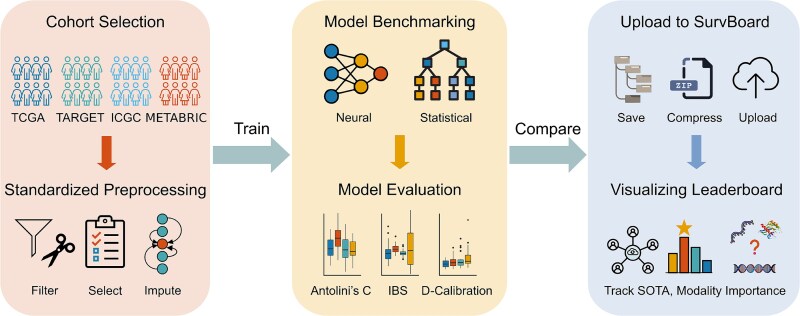
The SurvBoard framework enables the reproducible, easily accessible, and standardized comparison of (multi-)omics cancer survival methods. SurvBoard is based on a careful cohort selection from four cancer programs: TCGA, ICGC, TARGET, and METABRIC. The datasets from all programs are preprocessed in a standardized manner, which allows for uniform assessment of the models created. The evaluation results can then be uploaded to the SurvBoard leaderboard to track model results.

We developed a benchmark framework named SurvBoard that allows for the thorough evaluation of multi-omics survival models in the context of cancer. Our framework, SurvBoard, has several unique features (Fig. 1). First, we used datasets from four different cancer programs, TCGA, ICGC, TARGET, and METABRIC, containing data from up to seven modalities including clinical variables, gene expression, somatic mutations, DNA methylation, copy number alterations, protein expression from RPPA, and miRNA expression (Supplementary Tables S1 and S2, Methods, Supplementary Methods).

Second, the datasets were filtered and preprocessed in a standardized manner to enable optimal comparability across models.

Third, we prepared the datasets for conducting experiments in three different settings: (i) a standard setting, where the samples contain all modalities used in training, and the model training is performed individually on each cancer type, (ii) a missing-modality setting, where samples that are missing certain modalities are also included in training, and (iii) a pan-cancer training setting, where multiple cancer types are trained jointly via a unified model (Materials and Methods).

Last, we assessed the model performance using three different metrics that focus on the accuracy of patient outcome prediction and model calibration: Antolini’s C, IBS, and D-CAL (Methods, Supplementary Methods, Supplementary Table S3).

### Leaderboard

**Figure 2:**
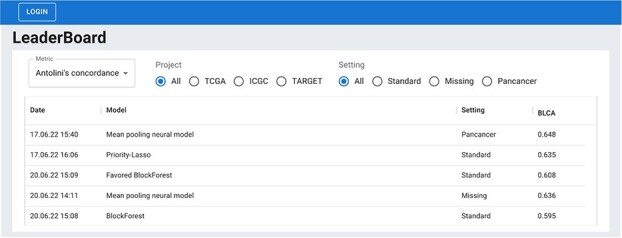
The SurvBoard web service curates and makes model results submitted to SurvBoard easily explorable and downloadable. The web service also ensures that SurvBoard stays up to date, as other researchers can easily extend our initial baseline models. A video tutorial is available on YouTube at https://youtu.be/HJrdpJP8Vvk.

We have developed a web service that enables researchers and other stakeholders to submit predictions on the SurvBoard benchmark set, which can be accessed via https://www.survboard.science/. Using this service, one can also download and inspect previous submissions, including our provided baselines. SurvBoard’s web service evaluates submitted predictions and displays the performance metrics for all datasets within the benchmark in an easy-to-compare leaderboard format (Fig. 2). To access a sample submission file and links to our web service, users may visit our GitHub repository. A video tutorial on how to use the Survboard leaderboard is available on YouTube at https://youtu.be/HJrdpJP8Vvk.

We seeded SurvBoard by submitting twelve models: two statistical and ten deep learning models trained on various combinations of input modalities and in different settings (Materials and Methods). We limited the selection of statistical models to those that had already demonstrated top performance in multi-omics cancer datasets in previous benchmarks [23, 25] (Materials and Methods). Hyperparameters of all methods were tuned using method-specific appropriate hyperparameter grids (Supplementary Methods).

### Assessment of model performances

To be fair to each model and to evaluate OS prediction performance, we first determined on which combination of modalities each model performed the best. We trained each model on each available modality (a) unimodally, (b) the combination of clinical data and gene expression, and (c) combination of all available modalities. The state-of-the-art deep learning models were applied only on (b) and (c), as their application on unimodal data was not meaningful. We selected the best modality set based on Antolini’s C metric for each model (Materials and Methods). We found that, broadly, clinical variables and gene expression data were the most predictive modalities across all models for both Antolini’s C and the IBS (Supplementary Figs S1 and S2).

**Figure 3:**
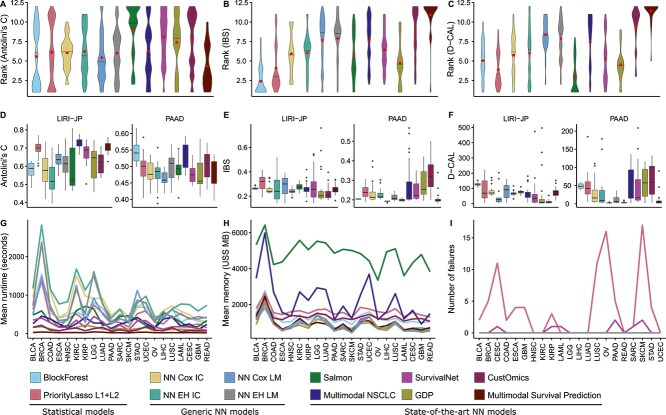
Regularized linear models and random-forest-based methods outperformed deep learning methods on the SurvBoard benchmark when trained on clinical and gene expression data, which was the most predictive modality combination across methods (Supplementary Figs S1 and S2). (A–C) Performance in the standard setting. Lower ranks indicate better performance. (A) Antolini’s C (discrimination). (B) Integrated Brier Score (IBS; discrimination + calibration). (C) D-Calibration (D-CAL; calibration). (D–F) Performance on external validation: models were trained on one dataset and tested on another of the same cancer type. (D) Antolini’s C. (E) IBS. (F) D-CAL. (G–I) Computational cost and model robustness. (G) Mean runtime as measured in seconds per cancer and method. (H) Mean memory usage (USS MB). (I) Number of model failures (e.g. numerical errors or fully sparse fits) across 25 folds. For failed folds, a KM estimator was used as fallback. NN: neural network.

Next, we evaluated the performance of each model on clinical data and gene expression sets using three performance metrics: Antolini’s C, IBS, and D-CAL. We observed that overall, BlockForest and PriorityLasso L1+L2 trained on clinical variables and gene expression data performed the best among all models (Fig. 3A–C). Notably, BlockForest achieved the best rank across datasets for IBS, and was among the better performers for Antolini’s C and D-CAL. Prioritylasso L1+L2 performed similarly well, achieving the second best rank for D-CAL and IBS, while also being among the top performers for Antolini’s C. For Antolini’s C specifically, Multimodal Survival Prediction [15] emerged as the top performer. However, differences among methods were less pronounced for this metric, with several models achieving very similar ranks.

Discriminative survival model prediction performance as measured by Antolini’s C was noticeably more concordant between deep learning methods than between PriorityLasso L1+L2 and BlockForest, or either of these models and any of the deep learning methods (Supplementary Fig. S3). However, overall, deep learning methods could not compete with the statistical models PriorityLasso L1+L2 and BlockForest. While Multimodal Survival Prediction performed well for Antolini’s C, achieving the best mean rank, it performed poorly in terms of model calibration, scoring one of the worst median ranks for both the IBS and D-CAL. Other deep learning-based methods performed similarly, achieving overall worse results than PriorityLasso L1+L2 and BlockForest in terms of IBS and D-CAL, while achieving variable results on Antolini’s C.

Results were largely concordant across projects, as exemplified by Antolini’s C; however, certain models performed particularly well on just one project. For instance, NN EH IC was the best-performing model on METABRIC but performed relatively poorly on ICGC (Supplementary Fig. S4). This variability is likely due to differences in sample size and input modalities.

We additionally investigated the generalization power of these models on an external dataset (Supplementary Methods), where each model was trained on one cohort and validated on a distinct cohort of the same cancer type (Fig. 3 D–F). The results varied by the cancer type and dataset size, showing no clear trend. For TCGA-PAAD, the BlockForest method performed the best, achieving the second highest Antolini’s C and one of the lowest IBS scores. For ICGC-LIRI-JP, the results differed. PriorityLasso L1+L2, Multimodal Survival Prediction and Multimodal NSCLC had the highest Antolini’s C values, but while Multimodal NSCLC and Multimodal Survival Prediction also performed well in terms of the IBS, PriorityLasso L1+L2 was one of the worst performers in terms of this metric. Similarly, most deep learning models performed better in terms of IBS than in terms of Antolini’s C. The D-CAL results were notably worse in absolute terms, likely due to the use of an external dataset leading to miscalibration, making it difficult to draw meaningful conclusions from this metric in the external validation scenario.

We compared the mean runtime and memory required to train all methods (Fig. 3G–H, Supplementary Methods). On average, in terms of runtime, across all cancer types, the statistical models required $499.73\pm 294.94$ s and the deep learning models required $327.87 \pm 280.91$ s to complete the training without parallelization. Multimodal Survival Prediction had the shortest runtime of $30.3 \pm 23.8$ s to complete training across all cancer types. The two classes of models showed similar statistics in terms of memory demands, with the statistical models requiring $1491.81 \pm 365.73$ MB compared to the deep learning models, which averaged $1430.52 \pm 1301.41$ MB. In our benchmark, only PriorityLasso L1+L2 and SurvivalNet had failures for some cancer types, primarily due to the Lasso yielding fully sparse fits for some cancer types for PriorityLasso L1+L2, and training instabilities occurring for some hyperparameter configurations for SurvivalNet (Fig. 3I). Overall, we did not find any notable differences in terms of computational requirements across methods. We note that these factors were strongly influenced by the chosen method of hyperparameter tuning (Supplementary Methods) and thus, cannot be expected to generalize broadly.

Since deep learning models tended to underperform other methods, we investigated the potential impact of effective sample size, in particular the number of events per dataset, on the performance difference between deep learning and other models. When trained only on gene expression data (unimodal), deep learning methods underperformed the elastic net and random survival forest methods, especially for datasets with smaller effective sizes, somewhat improving their relative performance as the number of events per dataset increased (Supplementary Fig. S5).

Hereafter, we focused on selected models within each model class: BlockForest and PriorityLasso L1+L2 for statistical models, and NN Cox IC and NN EH IC for deep learning, as intermediate concatenation tended to perform best for deep learning models. Moreover, implementations of our NN IC models better enabled downstream ablations.

### Added value of the pan-cancer training and including additional training samples with missing modalities

**Figure 4:**
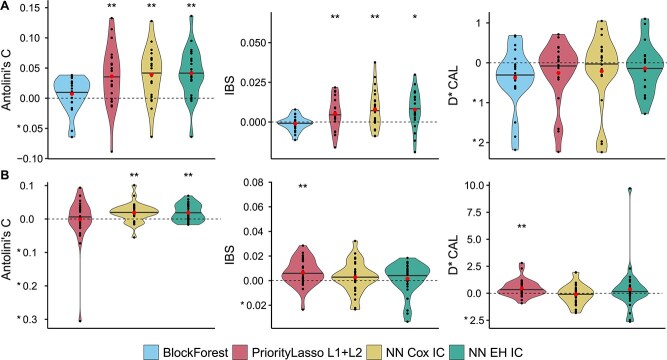
Pan-cancer training and inclusion of samples with missing modalities enhance performance across most models and metrics. (A) Effect of training models on multiple cancer types simultaneously (pan-cancer setting) using clinical and gene expression data measured as an absolute improvement of the metric score. Pan-cancer training significantly improved performance across two out of three metrics—Antolini’s C and IBS—for most models, compared to training on single cancer types. (B) Training on all available modalities, including samples with missing modalities, improved Antolini’s C for all deep learning methods considered, and both the IBS and D-CAL for PriorityLasso, compared to only training on samples without missing modalities. Significance indicated by ^*^ ($P <.05$) and ^*^^*^ ($P <.01$), based on a two-sided Wilcoxon signed-rank test against the standard setting. Black lines represent medians; red dots represent means.

Using the SurvBoard framework, we determined to what extent pan-cancer training, i.e. simultaneous training on all datasets from a cancer program, could improve the performance of omics survival analysis models. We used the two most informative modalities, clinical variables, and gene expression data, as input for the assessment. The results showed that pan-cancer training improved the median performance for most considered methods in terms of Antolini’s C and the IBS, while the impact on D-CAL was much more variable, and generally negative. Moreover, the performance increase was often statistically significant (Fig. 4A). Deep learning methods benefited the most from pan-cancer training, with all considered NN models significantly improving their performance for two out of the three considered metrics. Interestingly, however, the best-performing method in the standard setting, BlockForest, benefited the least from pan-cancer training.

Next, we investigated whether including additional samples with some missing input modalities during training could improve model prediction performance on unseen samples with all modalities present. For this, we performed experiments on all models capable of handling missing modalities (Supplementary Methods), namely all models from the pan-cancer setting except BlockForest. Since the clinical data and gene expression setting had no missing modality samples, we considered all available modalities and compared the performance of each method with and without including missing modality samples as additional training data. After including into the training set samples with missing modalities, Antolini’s C improved significantly relative to the non-missing modality models for all considered deep learning methods, but not for PriorityLasso L1+L2 (Fig. 4B). Meanwhile, only PriorityLasso L1+L2 showed significant improvement in model calibration as measured by IBS and D-CAL (Fig. 4B).

### Takeaways for effective model development and validation

In our benchmark framework, we have aimed to remedy potential pitfalls related to the training and validation of omics cancer survival models. The pitfalls discussed below emphasize the importance of some of the design choices we made in the SurvBoard benchmark and may be helpful for other researchers to validate their models on small $n$ and large $p$ data size regimes.

**Figure 5:**
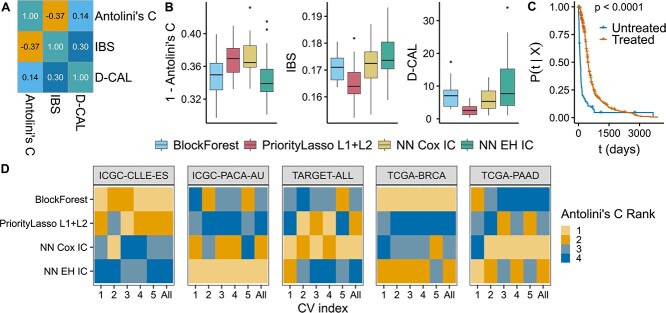
Pitfalls to consider when benchmarking and validating multi-omics survival models. (A) Pearson correlation matrix between Antolini’s C, IBS, and D-CAL of all models on breast cancer (METABRIC). The low correlation underscores the importance of using both discrimination and calibration metrics, as model rankings can differ substantially between them. (B) Performance of selected models on METABRIC, measured by 1—Antolini’s C, IBS, and D-CAL. Lower values indicate better performance. PriorityLasso L1+L2 achieved the best scores for both D-CAL and IBS but was among the worst for Antolini’s C, demonstrating the need for balanced metric reporting. (C) KM curves for glioblastoma multiforme (TCGA), stratified by radiation therapy status. Inclusion of treatment-related variables can introduce data leakage, as treatment decisions may depend on a patient’s prognosis [31]. Patients with unknown therapy status were excluded. (F) Ranks of models based on Antolini’s C across repeated cross-validation splits. Rankings varied substantially across repetitions, especially in small datasets (e.g. TARGET-ALL), illustrating the necessity of repeated cross-validations for robust performance estimation.

First, it is essential to report both discriminative and calibration metrics while evaluating survival models. Discriminative metrics such as Harrell’s concordance and Antolini’s C have been widely used, along with calibration metrics such as the IBS. These metrics do not necessarily correlate, with correlations close to zero or even negative on some datasets (Fig. 5A) [49]. Thus, reporting at least one metric of each type is crucial. We found that on various datasets included in SurvBoard, models could be favored if only one metric was reported. For example, on the METABRIC breast cancer dataset, PriorityLasso outperformed all other models in terms of the IBS and D-CAL while achieving among the worst concordance values as measured by Antolini’s C out of all methods (Fig. 5B).

Second, when using multi-omics survival methods, the choice of clinical variables is crucial for ensuring high performance. However, although it is tempting to use all treatment-related and outcome-related covariates as predictive features, including some of these may lead to data leakage [31]. For instance, a clinician might decide against starting radiation therapy if the patient is expected to have a short life expectancy due to their illness or other factors [50]. Indeed, on TCGA, we observed cancer types in which treatment-related variables such as radiation therapy were strongly associated with the outcome (Fig. 5C), which could be either due to a treatment effect or an effect of not prescribing the treatment due to a very advanced disease stage. It is thus advisable to be mindful of the choice of clinical variables, especially treatment variables, when benchmarking survival prediction methods.

Third, it is prudent to include an as large spectrum of studies and datasets as possible in a benchmark to account for variability in model performance. In SurvBoard, we reported results on datasets from four cancer programs. This may guard against overfitting to a particular cancer program that is frequently used in the literature or related work.

Fourth, one should avoid using unrepeated cross-validation or even a single split when reporting model prediction performance since this can make model ranking inconsistent. Indeed, in SurvBoard, we observed large variability in model rankings as measured by Antolini’s C across cross-validation repetitions for selected models (Fig. 5D). Smaller datasets, as measured by the number of events $e$, rather than the number of samples $n$, such as the Acute Lymphoblastic Leukemia (TARGET-ALL) dataset, were especially prone to this issue. Performance results on larger datasets tend to show greater consistency but may still suffer from sizeable variability. Thus, we suggest performing several repetitions of cross-validation on each dataset, ideally as many as is computationally feasible.

Fifth, it is crucial to ensure the comparability of past and future work. For example, when utilizing samples with missing modalities or training models on multiple cancer types, it is imperative to choose train and test splits that can also be utilized by models not utilizing these settings (for example, samples with missing modalities should not be part of the test set since this makes comparison with non-missing modality models impossible). To circumvent this issue, SurvBoard employed missing modality samples as additional training data instead of incorporating them into the test sets.

Finally, in addition to making the code for models publicly available, it is vital to focus on providing a reproducible hyperparameter tuning strategy and evading manual hyperparameter optimization to enhance the model’s reusability by other researchers.

## Discussion

In this work, we presented SurvBoard, a rigorous benchmark and a framework for the validation and comparison of omics survival models. In a proof of concept, here, SurvBoard enabled the comparisons of twelve models across 28 datasets from four projects. SurvBoard focused on model comparability by ensuring that models utilizing pan-cancer data or samples with missing modalities can be compared to models trained on single datasets. Additionally, we provided a simple web service that allows researchers to evaluate their models on our new benchmark easily. In our work, we also illustrated potential pitfalls during the validation of omics survival models, highlighting the importance of the choice of clinical variables, the use of repeated cross-validation, and the display of results using several relevant performance metrics.

Our observations that statistical models often outperform deep learning ones for the survival prediction in cancer and that clinical variables and gene expression data constitute the two most informative modalities were consistent with our earlier work [25] and the work of Herrmann *et al.* [23] and others; however, the current analysis encompassed a broader array of datasets and cancer programs. We also showed how the SurvBoard benchmarking platform enables novel findings. We demonstrated the positive effect of pan-cancer training for most of the survival analysis models considered in our leaderboard and examined the effect of conducting training on samples with missing data modalities [15, 24, 26].

In our experiments, we found that BlockForest achieved the best overall performance in SurvBoard. While some deep learning-based methods obtained more favorable Antolini’s C values, they performed considerably worse on metrics measuring calibration, such as the IBS and D-CAL. PriorityLasso L1+L2, a linear model, also ranked among the top performers, particularly excelling in calibration metrics such as IBS and D-CAL. Thus, in terms of the model class, there was no clear benefit to accounting for non-linearities or interactions in multi-omics survival analysis based on SurvBoard. This finding is perhaps unsurprising given the low sample sizes and high dimensionality of most datasets (Supplementary Table S1), where simpler models may generalize better. Additionally, linear models such as PriorityLasso L1+L2 offer the benefit of interpretability, which is often critical in biomedical settings. While recent work has been exploring approaches for improved interpretability of deep learning and random-forest-based methods in survival analysis and bioinformatics more generally, this point still stands in favor of simpler statistical methods [51–53].

In the future, further investigation is required to explore new model architectures and loss functions, particularly those that have been previously used in clinical survival analysis datasets [3, 5]. Methodological research could also focus on the problem of low model resistance to the inclusion of less informative data modalities [25]. Indeed, many methods continued to perform worse in our benchmark when more modalities were added, as evidenced by the fact that no models reached their best performance for most of the metrics when all modalities were included (Supplementary Figs S1 and S2). We also note that within SurvBoard, we restricted the benchmarking to methods that had been developed or previously applied in the multi-omics survival setting. It may also be interesting to adapt state-of-the-art methods for tabular data, such as XGBoost for survival data, to the multimodal setting [54].

It is important to consider some limitations of our work. While SurvBoard uses datasets from multiple cancer programs, the TCGA program contributed significantly more datasets than the others. Therefore, the conclusions drawn from smaller projects, such as METABRIC, TARGET, and ICGC, may be less reliable in comparison to those drawn from the larger TCGA dataset. Furthermore, when evaluating the overall results (i.e. pooled across all projects), the conclusions are still primarily influenced by TCGA, due to its large number of datasets. We also did not include datasets that did not meet the minimum requirements in terms of overall cohort size and the number of events per dataset to ensure meaningful metric calculation and model training. This practice may bias SurvBoard against cancer types for which it is not possible to commonly acquire large cohort sizes. We also acknowledge that deep learning methods may have underperformed, in part, because we used a pre-existing hyperparameter grid from Zhong *et al.*, adapting it only slightly to ensure numerical stability [4]. Thus, further fine-tuning of hyperparameters of deep learning methods may further improve their performance in future work.

Additionally, certain design decisions made during SurvBoard development may have an impact on the experiment outcomes. In particular, results might have varied if different design decisions had been made [30]. Nevertheless, we argue that defining a common benchmarking set for multi-omics survival analysis models is crucial to enable comparability across models, even if the benchmark has certain biases. Indeed, several standard computer vision datasets have recently been found to contain label errors and other inaccuracies. Despite these errors, these datasets have arguably greatly contributed to the progress of method development in that field [55]. One potential issue of SurvBoard is the approach used to fill in missing values for variables with missing data (Supplementary Methods). Similar to previous studies, we filled in these values in both the training and test sets together [22] such that researchers could focus on their primary objective of multi-omics survival analysis without the added concern of managing missing values. While data leakage, in general, can be dangerous [31], we believe that the impact of imputation in our case is minimal, and it prevents discrepancies in imputation methods across different studies [22, 41]. Furthermore, to ensure model comparisons in the pan-cancer setting, our datasets only included standard clinical variables such as demographics or staging, which may be a disadvantage to methods that heavily rely on clinical data.

Currently, to the best of our knowledge, multi-omics survival analysis methods are not yet commonly applied in clinics. Despite this, there could be potential future clinical implications of some of the methods considered here, in particular pan-cancer-based training. Models trained on pan-cancer data may benefit not only from a larger sample size but also from learning survival patterns across cancer types, leading to potentially more accurate survival probability predictions, particularly for rare or understudied cancers.

To sum up, the development of consistent preprocessing pipelines and online resources for evaluating multi-omics survival models is crucial to advancing research in the field of cancer. In the future, we expect our benchmarking framework to lead to more reliable conclusions about the superiority of different models in predicting patient survival.

**Key Points**

- We introduce SurvBoard, a comprehensive benchmarking framework for the standardized evaluation of multi-omics cancer survival models. SurvBoard provides an easily accessible platform for reproducible comparison of models trained on single-cancer and pan-cancer datasets. The platform addresses issues such as the impact of missing modalities and variability in experimental setups. SurvBoard integrates data from four major cancer programs–TCGA, ICGC, TARGET, and METABRIC–to ensure a comprehensive evaluation across diverse cancer types and research centers.
- SurvBoard results confirm that statistical models generally outperform deep learning models in survival function calibration. We also find that pan-cancer training improves model performance and that models benefit from incorporating data with missing modalities.
- SurvBoard includes a web service that allows researchers to submit models for benchmarking and evaluation. A leaderboard is accessible through https://survboard.science/ to promote transparency and the continuous assessment of models’ performance.

## Supplementary Material

Wissel_Supplementary_Materials_bbaf521
